# News Coverage of the COVID-19 Pandemic on Social Media and the Public’s Negative Emotions: Computational Study

**DOI:** 10.2196/48491

**Published:** 2024-06-06

**Authors:** Hanjing Wang, Yupeng Li, Xuan Ning

**Affiliations:** 1 School of Communication Hong Kong Baptist University Hong Kong China (Hong Kong); 2 Department of Interactive Media Hong Kong Baptist University Hong Kong China (Hong Kong); 3 Department of Social Sciences Beijing Normal University-Hong Kong Baptist University United International College Zhuhai China

**Keywords:** web news coverage, emotions, social media, Facebook, COVID-19

## Abstract

**Background:**

Social media has become an increasingly popular and critical tool for users to digest diverse information and express their perceptions and attitudes. While most studies endeavor to delineate the emotional responses of social media users, there is limited research exploring the factors associated with the emergence of emotions, particularly negative ones, during news consumption.

**Objective:**

We aim to first depict the web coverage by news organizations on social media and then explore the crucial elements of news coverage that trigger the public’s negative emotions. Our findings can act as a reference for responsible parties and news organizations in times of crisis.

**Methods:**

We collected 23,705 Facebook posts with 1,019,317 comments from the public pages of representative news organizations in Hong Kong. We used text mining techniques, such as topic models and Bidirectional Encoder Representations from Transformers, to analyze news components and public reactions. Beyond descriptive analysis, we used regression models to shed light on how news coverage on social media is associated with the public’s negative emotional responses.

**Results:**

Our results suggest that occurrences of issues regarding pandemic situations, antipandemic measures, and supportive actions are likely to reduce the public’s negative emotions, while comments on the posts mentioning the central government and the Government of Hong Kong reveal more negativeness. Negative and neutral media tones can alleviate the rage and interact with the subjects and issues in the news to affect users’ negative emotions. Post length is found to have a curvilinear relationship with users’ negative emotions.

**Conclusions:**

This study sheds light on the impacts of various components of news coverage (issues, subjects, media tone, and length) on social media on the public’s negative emotions (anger, fear, and sadness). Our comprehensive analysis provides a reference framework for efficient crisis communication for similar pandemics at present or in the future. This research, although first extending the analysis between the components of news coverage and negative user emotions to the scenario of social media, echoes previous studies drawn from traditional media and its derivatives, such as web newspapers. Although the era of COVID-19 pandemic gradually brings down the curtain, the commonality of this research and previous studies also contributes to establishing a clearer territory in the field of health crises.

## Introduction

### Background

The COVID-19 pandemic was one of the most unprecedented global crises in human history. Researchers have been groping for direct countermoves to the COVID-19 disease since its initial occurrence, with massive collaborative efforts committed. Notwithstanding, the pandemic posed evolving challenges to public health, politics, the economy, and personal well-being. The uncertainty that comes along with pandemics greatly disturbed people’s current lives and changed their expectations for the future. The public health situation in Hong Kong was once severe, with >10,000 new confirmed cases on average being reported each day during the fifth wave of the COVID-19 pandemic [1]. It is astonishing that Hong Kong ranked the highest fatality rate in the world given its first-class medical care quality [2].

Pandemics usually bring negative emotions, such as anger, sadness, fear, etc [3]. Before the rebound of the pandemic, Hong Kong had been expecting the border to reopen with mainland China after rounds of negotiations as proof of efforts to keep the pandemic situation under control. The suddenly acute situation rapidly crashed people’s dreams of reuniting with family or regular economic exchanges, making them feel at sea, along with the unavoidable increased level of depression [4,5]. It is also reported that citizens panic bought daily necessities amid the possibility of a citywide lockdown [6], when a substantial number of residents experienced anxiety, loneliness, and even mental health issues [7]. The negative feelings of individuals, such as fear, anger, and sadness, which are bound to impair people’s state of mind, call for close attention because they will affect people’s cognitive as well as their physical health [8]. When it comes to communities, the collective sentiment is also devoted to shaping the distribution of formal social control measures, which is an important expression of the collective sentiment [9]. The negative emotions, if constantly diffused, could contribute to the evolution of a societal negative emotional climate, which might lead to changes in personal values and further political consequences and normative implications [10].

The primary and authoritative source that informs citizens of pandemic situations and public health responses is news media, which is an institutionalized channel that broadcasts crisis information to a broad audience and is an inseparable part of political crisis management [11,12]. Meanwhile, the internet offers a capacious playground for official news accounts to play their roles while meeting the goal of being timely. Among the digital organisms that rejuvenate news media, social media is a representative information carrier for content consumption during crises [13]. News consumption on social media has been a prevalent practice for audiences to establish connections with the uncharted world, thereby affecting their understanding of news subjects and events unconsciously. This is particularly true for those who are under quarantine and obeying social distancing rules during the urgent level of a pandemic. When web news coverage of a crisis triggers the audience’s emotions, the collective sentiment concerning cultural, social, political, or economic events can be directly observed via user interactions on the same platform. Hence, the investigation of news coverage on social media may directly enable the news media to play a better role as a gatekeeper in crisis communication.

Most of the COVID-19–related research on web-based emotional expressions focuses on describing components and intensity instead of attempting to explain the reason for the emergence of the emotions [14]. Specifically, we have limited systematic knowledge of the connection between crisis coverage on social media and users’ emotionality [15]. Moreover, existing studies usually set aggregate emotionality (ie, the sum of all the specific emotions) as their target instead of focusing on the negative emotions, which are worth extra attention. A deeper understanding of emotional responses that might deteriorate mental health is crucial for the well-being of individuals caught in a crisis. Besides, negative emotions and positive emotions amid a crisis function oppositely regarding perceptions; for example, the former may foster a greater perceived threat and can contribute to far-reaching impacts on individuals, such as their shift in values [10]. Thus, we devoted our efforts to filling the identified research gap by endeavoring to explain the negative emotions of the audience from the perspective of news coverage on social media. Given our research context of Hong Kong, we chose to examine COVID-19–related news posts and their associated comments on Facebook, the most popular and widely used social platform in Hong Kong [16].

Accordingly, our study proposed two research questions (RQs):

- RQ1: what and how are COVID-19–related issues and subjects of web news coverage by official news accounts associated with negative emotions in Facebook users’ comments in Hong Kong?
- RQ2: what are the effects of media tone and the interaction effects of media tone with issues or subjects on negative emotions in Facebook users’ comments in Hong Kong?

In this regard, we crawled Facebook COVID-19–related posts published by representative Hong Kong news organizations and the corresponding comments to investigate how news coverage on social media affects the public’s negative emotions amid the fifth wave of the pandemic. We situated the examination of negative emotional responses and crucial news elements of subjects, issues, media tone, and length in the framework of appraisal theory. Specifically, we first used seed words, such as “COVID-19,” “coronavirus,” “Omicron,” etc, to filter out the irrelevant, that is, not COVID-19–related, posts and identify other related words for post selections. Afterward, we managed to locate the focal points of societal attention under the threat of COVID-19 through frequent words and topic models, and we mapped keywords to identify issues and subjects. Then, we leveraged state-of-the-art machine learning techniques to reveal the emotion dynamics and sentiment embedded in the news posts. Finally, we used regression analysis with fixed effects to better understand and explain users’ negative emotional responses.

### Research Framework and Hypotheses

#### Overview

**Figure 1:**
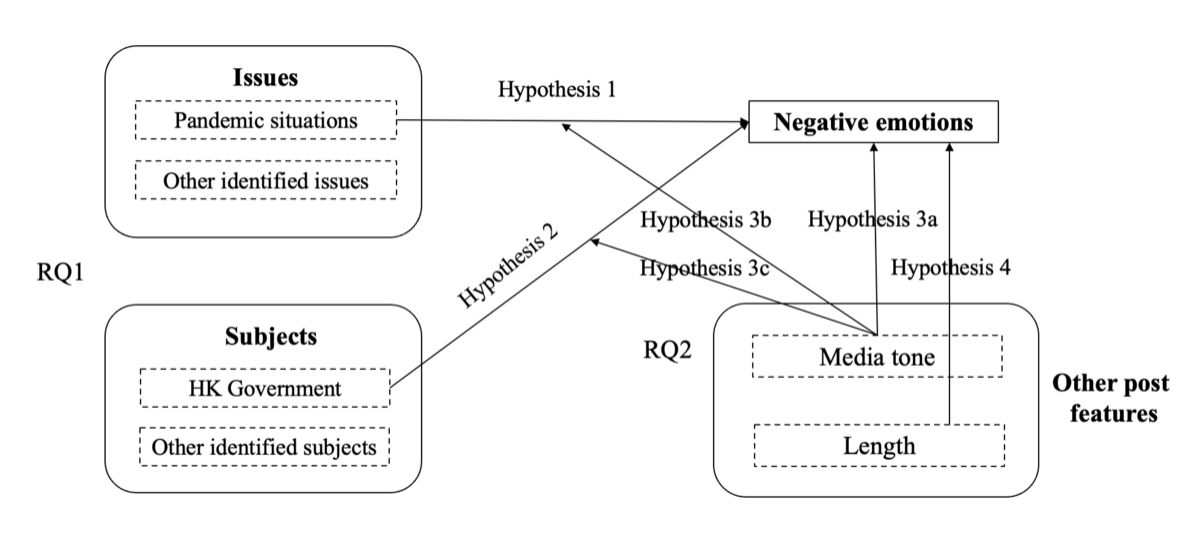
Theoretical framework. HK: Hong Kong; RQ: research question.

We situated our research framework in appraisal theory [17-19]. Our motivation for applying appraisal theory to analyze web news coverage was that emotions emerge through the evaluative process of particular circumstances, eliciting distinct responses among individuals [20]. The appraisal theory laid the foundation for sentiment extraction and thus provided insights for understanding the emotions of social media posts, such as content created by microbloggers to explore the minds of authors [20]. In our paper, we constructed the examination of subjects and issues studied by previous studies [15], together with other identified crucial news elements, media tone, and length, on the basis of the framework of appraisal theory. We studied the appraisal expressions from the aspect of attitudes [21], that is, the negative emotions (anger, fear, and sadness) of the appraiser, that is, the audiences or the commenters of the news posts. We sought to develop a research model based on the effect of news coverage from the perspectives of issues, subjects, media tone, and news length on users’ negative emotions. We put forth each hypothesis after discussing the theoretical basis. Our research model is presented in Figure 1.

#### Issues and Subjects

According to appraisal theory, emotions are prompted based on subjective assessments of events and situations according to personal significance [18]. The elements of news coverage may arouse the feelings of the audience, and the individual differences in the appraisal of news content may be illustrated in variations of audience responses [22], which is also applicable in crisis communication [23,24]. Prior studies identified news topics and the content features per se as critical factors in explaining the engagement of commenting behavior [25]. Users that are aroused by the covered topics may desire to express their opinions or attitudes and thus have the motivation to comment with emotions [26,27]. Existing findings explore the different effects of news stories’ framing elements on readers’ responses. It is suggested that news topics such as political conflicts have more comments but also more toxicity, while topics such as science and technology induce fewer toxic comments [28,29]. Specific subtopics of health in web news are also found to contribute to different idiosyncratic reader responses [30]. Scholars have applied categorized issues and local political actors to analyze the web news coverage of websites in the early stage of COVID-19 [15].

In this study, drawing on appraisal theory, we intended to postulate the impacts of issues and subjects covered in news posts on the negative emotions in users’ comments, which refer to the mentioned important combined subtopics and the subjects in the posts, respectively. Specifically, we decided to examine the role of issues and subjects that are characterized as ubiquitously dramatic and evocative for a lasting period, which also received adequate attention from mainstream media [31]. On the basis of these criteria, one of the most representative and worldwide universal issues is pandemic situations [32-34], in which information such as confirmed cases and regional risk levels is presented. As for subjects, the government, which acts as a crisis manager, plays an essential role in crisis management [15]. The performance of the government in coping with the crisis may arouse public emotions [35,36], and the clues of government inefficiency in problem-solving are prone to stimulating negative feedback [25,37].

Hence, regarding RQ1, we proposed the following hypotheses:

- Hypothesis 1: the occurrence of the issue about pandemic situations in the COVID-19–related news post would be associated with a higher level of negative emotions in user comments.
- Hypothesis 2: the occurrence of the subject about the Government of Hong Kong in the COVID-19–related news post would be associated with a higher level of negative emotions in user comments.

#### Media Tone

The news coverage sometimes manifests a preferred tone selectively. Mass communication has priming effects on the general audience given the selective attention of people [38,39]. The evaluative tone of media coverage is conceptualized as a composition of second-level agenda setting [40]. People routinely draw upon particularly salient information, which is shaped considerably by mass media. Media tone provides us a more detailed understanding of incidents together with subsequent attitudes and opinions [41], public’s perceptions regarding the economy [42], or even influences observable behavior [39]. Research on economic news coverage suggests that newspapers exhibit a negativity bias while Twitter exhibits a positivity bias toward the same undesirable event [43]. The media tone may directly act on the readers as the message valence may affect the emotional and cognitive responses of audience members [44]. Multifarious studies have shown that the emotional valence of crisis media content has a significant impact on user engagement [45,46], but whether the conclusions are safely applicable to emotional responses is still in the darkness. Eisele et al [15] found that the negativity and positivity of web news articles both have limited effects on the emotionality of commenting; meanwhile, the contrasting evidence shows that stronger audience reactions often accompany negative news [47,48]. The audience’s bias may lead to asymmetrical effects on the audience under positive and negative tones; additionally, the latter could have polarizing power on the news audience [49] and a greater effect on message reception [50]. The negatively presented crisis may arouse pessimism. Moreover, the audience would blame an undesirable event in negatively framed news on the government [51].

With reference to RQ2, we formulated the following hypothesis:

- Hypothesis 3a: the negative tone of a COVID-19–related news post would be more likely to increase the level of negative emotions in user comments than a positive tone.
- Hypothesis 3b: the interaction effect of a negative tone in the COVID-19–related news post and the occurrence of the issue of pandemic situations would increase the level of negative emotions in user comments.
- Hypothesis 3c: the interaction effect of a negative tone in the COVID-19–related news post and the occurrence of the subject of the Government of Hong Kong would increase the level of negative emotions in user comments.

#### Post Length

Length is one of the crucial variables that characterize content [52], whose impacts on user engagement are often discussed [53,54]. Existing studies consider content length as one of the dimensions of reviewers’ perception and evaluation based on cognitive overload theory [55]. When individuals are experiencing information processing, sometimes not all the inputs can be fully digested and used, which refers to cognitive overload [56,57]. For the extrinsic load, which means the individuals are free of the inherent trouble with information processing, the load is induced by how the content is organized [58-60]. Prior studies have discovered that a total lack of text or overabundant text is inefficient in provoking engagement, in contrast to an appropriate text scale [55,61]. The extra cognitive load is also found to have positive effects on negative emotions, which suggests that the information contained within the content once exceeds the scope of proper readability would induce negativeness in readers [62,63]. We conceptualized length as a crucial news feature because the size of the news can affect the diversity of content, the thinking depth of the audience, and the attractiveness of the news [64,65]. Therefore, we developed a hypothesis as follows:

- Hypothesis 4: the length of the COVID-19–related news post would be curvilinearly related to the level of negative emotions in user comments.

## Methods

### Overview

We chose Facebook as the target social platform for our research for the following reasons. First, Facebook is one of the most influential and popular social media platforms globally and the most used platform in Hong Kong [16]. Facebook users are concentrated in the age group of 18 to 44 years, which accounts for more than half the population in Hong Kong [66]. In addition, news organizations in Hong Kong use Facebook as an integrated channel for publishing up-to-date news, self-promotion, and connecting with readers, which allows researchers to observe their interactions from public pages directly.

**Figure 2:**
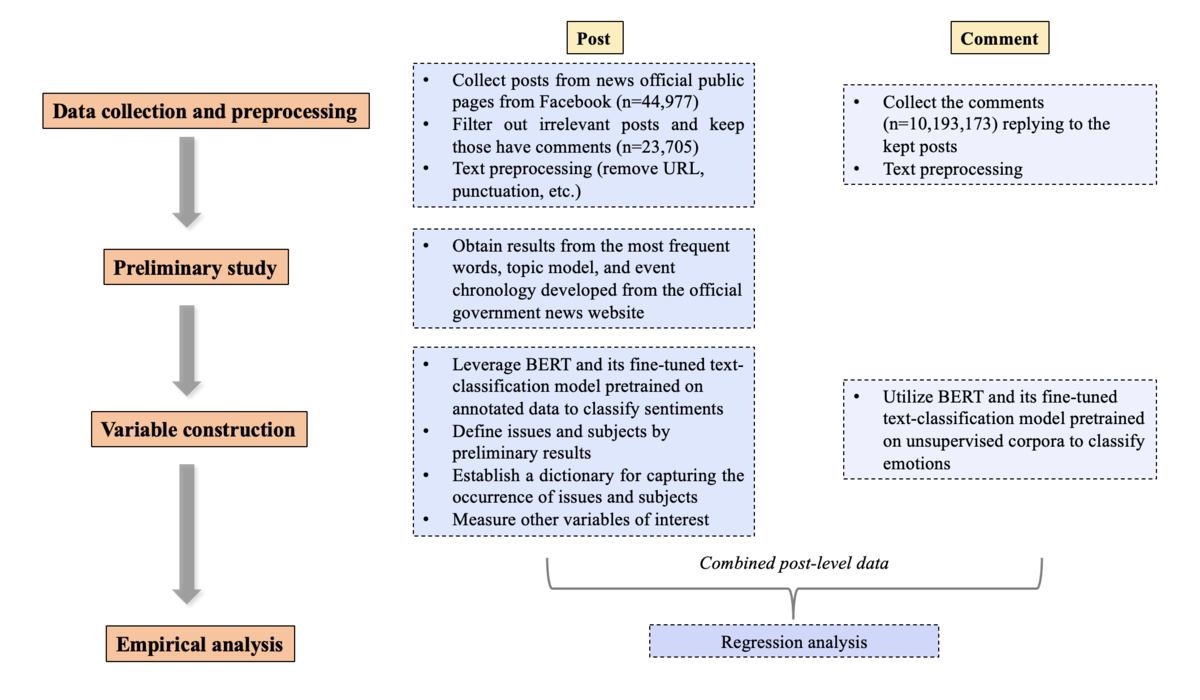
Analytical flow. BERT: Bidirectional Encoder Representations from Transformers.

For variable construction, we leveraged 2 fine-tuned models of Bidirectional Encoder Representations from Transformers (BERT) [67,68], which is considered the best-performing computational method [69], to classify the emotions revealed in user comments and the sentiment of the posts, respectively. For news coverage, we mainly applied the topic model for subtopics and references in dictionary development and then mapped the preprocessed news pieces into issues and subjects. Finally, we adopted negative binomial regression for data analysis. The measurements of variables were conducted in Python (Python Software Foundation), and the statistical analysis was implemented in RStudio (Posit). The analytical flow is shown in Figure 2.

### Data Collection and Preprocessing

To shed light on the emotional dynamics, we crawled posts and comments on Facebook. Specifically, we first determined the public pages of the major mainstream news accounts (refer to Table S1 in Multimedia Appendix 1), with about 505,371 (SD 364,386) followers on average. The collection period ranges from January 2022 to May 2022, which almost covered one complete circle of the fifth wave of COVID-19 in Hong Kong [70]. To filter the COVID-19–related posts, we developed a list of keywords, as shown in Textbox 1.

We started by searching with the seed words, such as “COVID-19,” “Omicron,” and “fifth wave,” that commonly occur in public view, and we added words of posts in the searching results that both concurred with the seed words and are directly related to the pandemic. The process would be repeated until the keywords list remained unchanged for an extra 50 posts. The posts that merely hit the keywords but were concerned about irrelevant issues, such as the promotion slogan of news organization apps, were left out. The comments on the filtered posts were collected correspondingly. The filtering criteria reached the agreement of all the coauthors. After filtering out those irrelevant posts, we collected 40,701 posts, of which 23,705 posts have 1,019,317 comments in total.

Before the measurement of variables, we first cleaned the texts for subsequent analysis. The messages on social platforms are usually presented informally, which might influence the machine learning model performance [71]; thus, text preprocessing is an essential prerequisite before the model training [72-74]. To clean the data, the posts and comments were processed by removing the URLs as well as redundant punctuation and normalizing the whitespaces. We then leveraged the OpenCC library [75] to convert simplified Chinese characters to Hong Kong standard traditional Chinese characters.

**Keywords for extracting COVID-19–related posts.**

**Original keywords**

- *Covid*, 疫苗, 新型冠狀病毒, 新冠, 疫情, *Omicron*, 奧密克戎, 抗疫, 快測申報, 檢測, 確診, 無症狀, 陽性, 防疫, 隔離, 禁足, 強制檢測, 方艙, 封城, 第五波, 快速檢測, 動態清零

**Translation**

- *Covid, vaccine, COVID-19, pandemic situation, Omicron, anti-pandemic, rapid diagnostic test (report), confirmed cases, asymptomatic infection, tested positive, pandemic prevention, quarantine, lockdown, compulsory testing, mobile cabin hospital, fifth wave, dynamic clearing*.

### Ethical Considerations

This study is a computational analysis that uses publicly available data, which do not include personally identifiable information or sensitive individual data. Our research complies with the principles of ethical conduct in research involving data without interaction with human subjects and identifiable private information. As such, the nature of this study did not necessitate the application for ethics board review or approval. All the data are securely stored and only accessed by personnel involved in the research team.

### Operation of Variables

#### Dependent Variable

**Figure 3:**
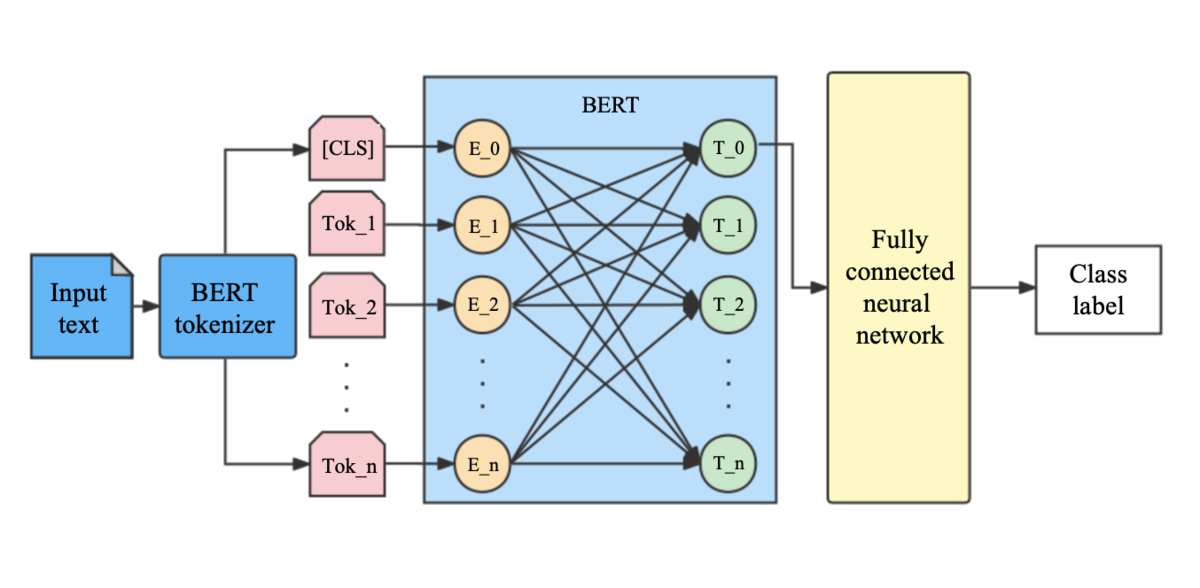
Analytical flow. BERT: Bidirectional Encoder Representations from Transformers. CLS: the special token “classification”.

Our dependent variable was the negative emotions conveyed by the user comments, which was measured by the aggregate number of comments labeled as negative emotions under each post. Automatic recognition of emotional information in social media texts based on natural language processing technology can help understand the attitude of netizens toward various events and portray people’s emotional fluctuations on a large scale over time. To this end, we adopted an effective method [76] to fine-tune a pretrained language model called BERT [67], which has proven to be empirically powerful in natural language processing. In carrying out super large-scale pretraining on unsupervised corpora, the fine-tuned BERT model adopts the transformer encoder as the language model and infers the relationship between input and output completely through the attention mechanism [68]. The model’s architecture is provided in Figure 3.

We trained the deep learning model on the SMP2020-EWECT COVID-19 pandemic Weibo data set [77]. We adopted the data set from Weibo because Hong Kong and the Chinese mainland share a generally similar sociocultural context. While the model was trained on a simplified Chinese data set, preliminary experiments show that models trained on simplified Chinese data sets can be directly used to infer Cantonese texts. The data set consists of the COVID-19–related microblogs since the outbreak of the COVID-19 pandemic, that is, the user-generated postings, on Weibo, which is one of the largest Chinese social media platforms. Each microblog has been previously labeled in the categories “happy,” “surprise,” “neutral,” “sad,” “fear,” and “angry,” and the last 3 categories of emotions belong to negative emotions. The split of the training set, validation set, and test set was predetermined to be around 8:2:3. We used microblogs in the training set for model training and the predicted results of the labeled microblogs for calculating cross-entropy loss. The evaluation of the test set suggested that the model can achieve an accuracy of 0.772 and a macroaveraged *F*_1_-score of 0.739, which has a better performance than classifiers of similar tasks [78]. Since the recent development of large language models, ChatGPT (OpenAI) [79] has achieved comparable performance compared with BERT in sentiment analysis [80], and we evaluated the performance of BERT and ChatGPT 3.5 and validated it by human assessment of random samples. Overall, BERT performed better than ChatGPT with an accuracy of 0.73 versus 0.48 on the basis of human assessment. The detailed assessment of the models is shown in Table 1.

The output of the emotion detection model applied to our collected data was one-hot encoded. In other words, if a comment is recognized as one kind of emotion, for instance, “angry,” the value of that comment under the category “angry” would be “1”; otherwise, it would be “0.” As our dependent variable was the negative emotions, we only focused on the emotions “angry,” “fear,” and “sad.” The number of emotions belonging to the comments under the same post were aggregated. The examples of 6 emotions and their average amount per post are presented in Table 2.

**Table 1:** Comparison of performance metrics of Bidirectional Encoder Representations from Transformers (BERT) and ChatGPT in emotion detection.

Model and emotion			Precision		Recall		*F*_1_-score
**BERT**							
	Angry	0.40		0.89		0.55	
	Sad	0.99		0.79		0.88	
	Fear	0.36		0.73		0.48	
	Neutral	0.96		0.51		0.66	
	Happy	0.80		0.86		0.83	
	Surprise	0.85		0.87		0.86	
**ChatGPT**							
	Angry	0.37		0.44		0.40	
	Sad	0.75		0.44		0.55	
	Fear	0.48		0.49		0.48	
	Neutral	0.42		0.69		0.53	
	Happy	0.68		0.39		0.49	
	Surprise	0.35		0.21		0.27	

**Table 2:** Examples of emotions and average number per post.

Emotion	Examples	Number per post, mean (SD)
Happy	“A thought on the wrong path, thankfully no misfortune has happened! The hard times will eventually pass, hang on!”	6.28 (67.84)
Angry	“With so much money spent on building cabins, is there a place for the homeless?”	24.13 (51.73)
Sad	“They are all poor people, so desperate that they have to rob, but the shopkeepers already barely make a living.”	0.83 (2.39)
Fear	“I’ll be scared to death if not die of sickness. I’m extremely anxious!”	1.57 (3.94)
Surprise	“Losing millions every month? Unbelievable”	0.69 (1.94)
Neutral	“Put more money into medical care. Focus on the elderly and children.”	10.95 (67.20)

#### Independent Variables and Control Variables

##### Issues and Subjects

As the pandemic evolution, policies, and terminologies are highly varied among countries and regions, there is no universally available or applicable taxonomy for us to map the news coverage of COVID-19 in Hong Kong on Facebook. Thereby, we applied the philosophy of grounded theory [81] to learn the structure of content and establish the foundation for the mapping of news coverage. To better grasp the overview of our post data, we first collected the frequent words of monthly posts and the total posts to locate those words that are consistent across months or time varying to have an initial understanding of our post data. We limited the frequent words to the top 50 words for consideration of relevance and representativeness after comparison. The results from comparison indicated that pandemic situations, testing, confirmed cases, vaccines, and economic consequences were the most outstanding and commonly shared words across months.

**Figure 4:**
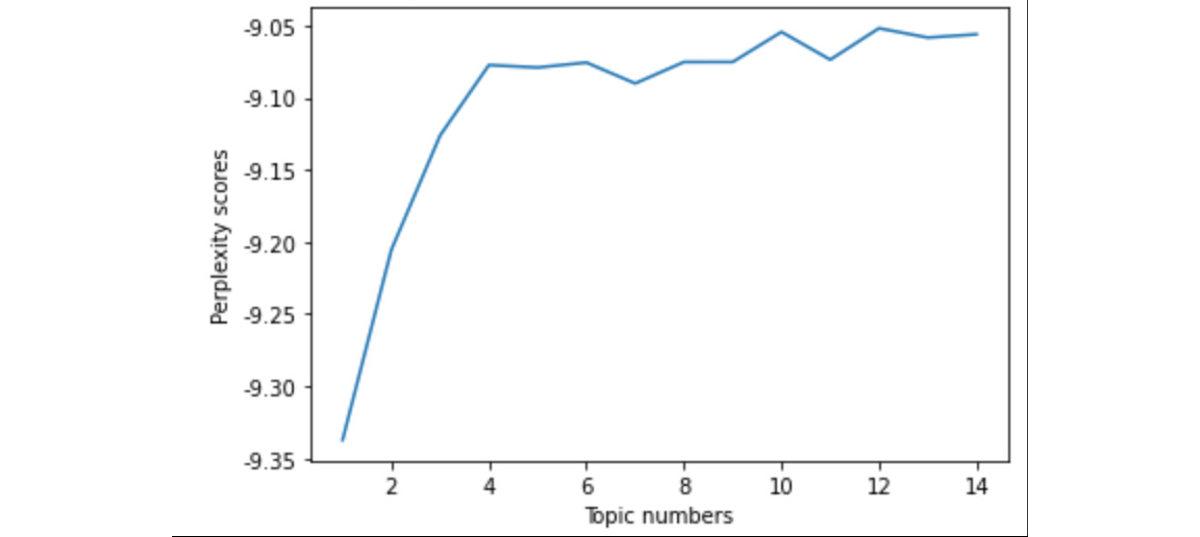
Perplexity scores under different numbers of topics.

**Figure 5:**
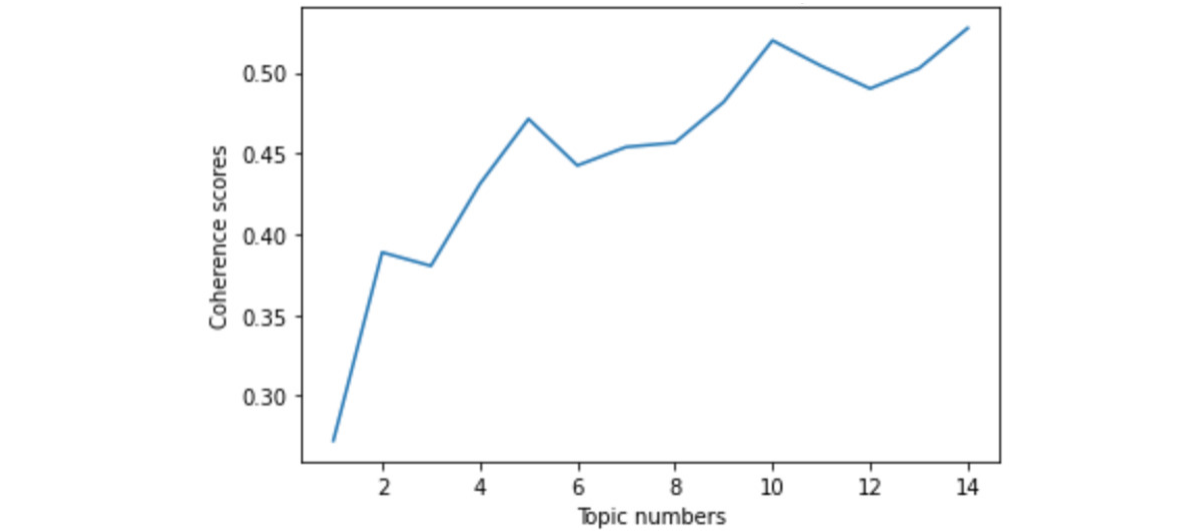
Coherence scores under different numbers of topics.

Then, to layout the topics for the preparation of the topics and subjects, we used the latent Dirichlet allocation topic model [82]. Latent Dirichlet allocation does well at discovering latent topics from given words and documents, which enables us to encode many different kinds of prior knowledge [83]. To determine the optimal number of topics, we calculated the perplexity and coherence scores of models with varied numbers of topics separately in Figures 4 and 5. A lower perplexity score and a higher coherence score suggest a topic model with better performance [84]. Taking the perplexity and coherence scores into account, we decided 8 to be the topic numbers in cases of overfitting issues. The topics along with their top words are shown in Table S2 in Multimedia Appendix 1. The output of our topic model divided the content into 8 subtopics, which required further inspection because we intended to disentangle the issues and the subjects that may intersect among the topics.

Afterward, we randomly sampled 50 posts each from the collected data of news organization accounts to identify categories of issues and subjects based on preliminary work, to which we constantly compared the posts and assigned them accordingly. We determined the categorization rubrics of issues according to two criteria: (1) each post is supposed to mention at least one of the identified issues and (2) the keywords of issues and the subjects should be mutually exclusive. The categorization of subjects was similar, but the posts may not necessarily mention any of the subjects. After defining the categories, we set up unique keywords for each issue and sector for labeling the posts. Aside from the data we acquired, we also developed a highlighted-event chronology from the Government of Hong Kong’s official news website [85] to help validate the process of establishing dictionaries and ensure the credibility of the construction of news coverage. The keywords were selected through initial screening based on findings from the most frequent words and topic model and then modified through rounds of validation on a random set of 50 posts each time. The adjustment to keywords was repeated until the dictionaries did not change further. The categories of issues and subjects with examples and times of occurrences are presented in Table 3.

**Table 3:** Examples and occurrences of the categorized issues and subjects.

Category			Examples		Occurrences, n
**Issues**					
	Pandemic situations	“Five members of the Cathay Pacific crew were infected with the Omicron variant virus.”		18,929	
	Supportive actions	“The sixth round of the AEF comprises 48 schemes which are estimated to benefit 67,000 businesses, operators of 40,000 transport tools and 750,000 individuals.”		1443	
	Information and advice	“The WHO does not consider the Omicron variant IHU to be a significant threat at this time.”		8596	
	Antipandemic measures	“In view of the rapidly worsening global pandemic situation due to the Omicron variant, passenger flights from eight countries will not be permitted to land in Hong Kong from January 8.”		12,523	
**Subjects**					
	Government of Hong Kong	“Chief Executive: surrendering to the virus is not an option.”		7992	
	Central government	“The Leader of the Mainland Chinese medicine expert group of the Central Authorities, Mr Tong Xiaolin, today led a delegation of Chinese medicine experts from the Mainland to Hong Kong for field visits and offering support in Hong Kong’s fight against the pandemic.”		1808	
	Functional sectors	“The Hospital Authority said its main strategy for adjusting some nonemergency services is to safeguard the public.”		1795	

##### Media Tone

To determine the sentiments of posts, we applied the data set of sentiment detection of Weibo users during the COVID-19 pandemic [86] to fine-tune a BERT-based text classification model for sentiment analysis. The data set includes 1,000,000 Weibo posts from January 1, 2020, to February 20, 2020, and 10% of them were previously labeled with 1 (positive), 0 (neutral), and −1 (negative). The model architecture for media tone detection is similar to that in Figure 3 to infer the sentiment of the collected posts. It turns out that COVID-19 coverage on social media in Hong Kong was dominated by a neutral tone, with 226 posts with negative tone and 461 with positive tone. The detailed assessment of model metrics is listed in Table 4.

**Table 4:** Comparison of performance metrics of Bidirectional Encoder Representations from Transformers (BERT) and ChatGPT in sentiment analysis.

Model and tone		Precision	Recall	*F*_1_-score
**BERT**				
	Positive tone	0.72	0.96	0.82
	Negative tone	0.97	0.95	0.96
	Neutral tone	0.96	0.78	0.86
**ChatGPT**				
	Positive tone	0.57	0.87	0.69
	Negative tone	0.67	0.94	0.78
	Neutral tone	0.76	0.26	0.39

##### Length

We used Python to measure the length of the string of post content; in other words, the number of words each post contains is treated as the numerical value of the variable length (mean 212.44, SD 152.24).

Besides, extant studies found a rise in negative emotional responses with the increase in COVID-19 cases [87], so we controlled the *daily confirmed cases* to indicate the severity of the pandemic at the time of posting, which is the number of new COVID-19 cases within the same day [88]. We also controlled the *fixed effects of the posting accounts of news organizations* in the form of dummy variables to control for variables that are not observable or measurable across the accounts [89,90] and *the fixed effects of time by adding dummy variables for months in the regression models* [55,91].

### Data Analysis

Considering that most of our variables are dummy variables and categorical variables, we used the negative binomial model, which is suitable for modeling count data with overdispersion and is commonly applied in social media research [92-94]. To examine the conditions for the negative binomial model, we first calculated the ratio of residual deviance and its *df* in a Poisson regression model, which was 35.657 (much greater than 1). Moreover, we calculated the variance inflation factors in a multiple regression model to examine the multicollinearity [95]. As Table S3 in Multimedia Appendix 1 shows, the variance inflation factors of all the variables were not unusually >1.0, which means there is no concern for multicollinearity. Consequently, we chose the negative binomial regression on postlevel data to explore the characteristics that are supposed to explain the negative emotions revealed in the comments. All the regression analyses were conducted using the package *MASS* [96] in RStudio.

## Results

### Descriptive Summary

**Figure 6:**
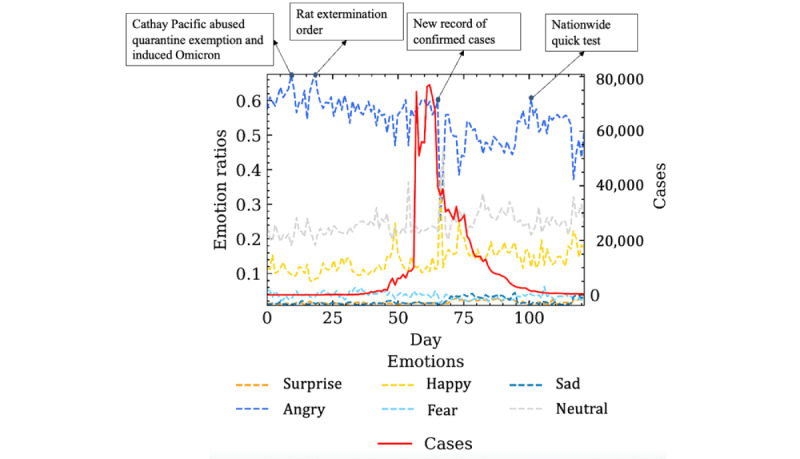
Emotion dynamics of detailed categories and daily confirmed cases.

Figure 6 illustrates the temporal dynamics of each type of emotion. The dashed lines represent each emotion, while the solid line represents the daily confirmed cases. Overall, anger occupied the largest proportion of all emotions. From the selected events labeled in Figure 6, we can tell that the temporary peaks of the negative emotions coincided with the representative events we identified from the aforementioned work of event chronology and distinct terms of the most frequent words. The exploratory findings demonstrated that users’ negative emotions may be aroused by particular events.

### Regression

Aside from the dummy variables, we normalized the rest of the variables because of their differentiated magnitudes. We applied R (version 4.1.2; R Foundation for Statistical Computing) to implement data analysis. Table 5 depicts the statistics of regression, in which we tested the effects of issues, subjects, and other content characteristics of news posts on the users’ negative emotions.

With the fixed effect of publishing account and months controlled, we first included the main variables of interest in model 1 (ie, issues, subjects, and media tones), then we added the interaction effects of media tone (ie, media tone×Government of Hong Kong and media tone×pandemic situation) in model 2, and we finally added squared variables (ie, squared terms of length and case number) in model 3.

The salience of issues in news coverage reflected the primary concerns of the citizens facing the pandemic crisis. Among the 4 issues, antipandemic measures (*B*=–0.050; *P*=.006), pandemic situations (*B*=–0.261; *P*<.001), and supportive actions (*B*=–0.083; *P*=.02) had a significant negative influence on the negative emotions; that is, the occurrence of these 3 issues could decrease the log count of negative user emotions. The effect of the issue on information and advice, although not significant and minuscule (*B*=0.005; *P*=.80), illustrated a positive impact on the log count of negative emotions. The prominent subjects, on the other hand, demonstrated those whom the public considers to play a crucial part in crisis administration. Both the governments of the Chinese mainland (*B*=0.120; *P*<.001) and Hong Kong (*B*=0.324; *P*<.001) had positive impacts on users’ negative emotions. Besides, the results illustrated that the existence of functional sectors had a negative effect, although not that significant, on negative emotional responses (*B*=–0.058; *P*=.09).

Compared with the positive tone embedded in the news posts, the neutral tone (*B*=–0.338; *P*<.001) and negative tone (*B*=–0.496; *P*<.001) had negative effects on the log count of negative emotions. In other words, the neutral sentiment and negative sentiment manifested in posts would both decrease audience’s negative emotions. This finding may be due to the backfire effects of the out-of-place intentional positive media tone amid the dire pandemic situation. Similar effects appear in the processes involved in the forced declaration of preferred pronouns, ensuring the persistence of public opinion by an authority’s commands [97], and the perception of corporate reputation in response to promotional tone [98] in that the audience tends to lack trust in the sincerity of such commitment and negative reactions emerge subsequently [99].

**Figure 7:**
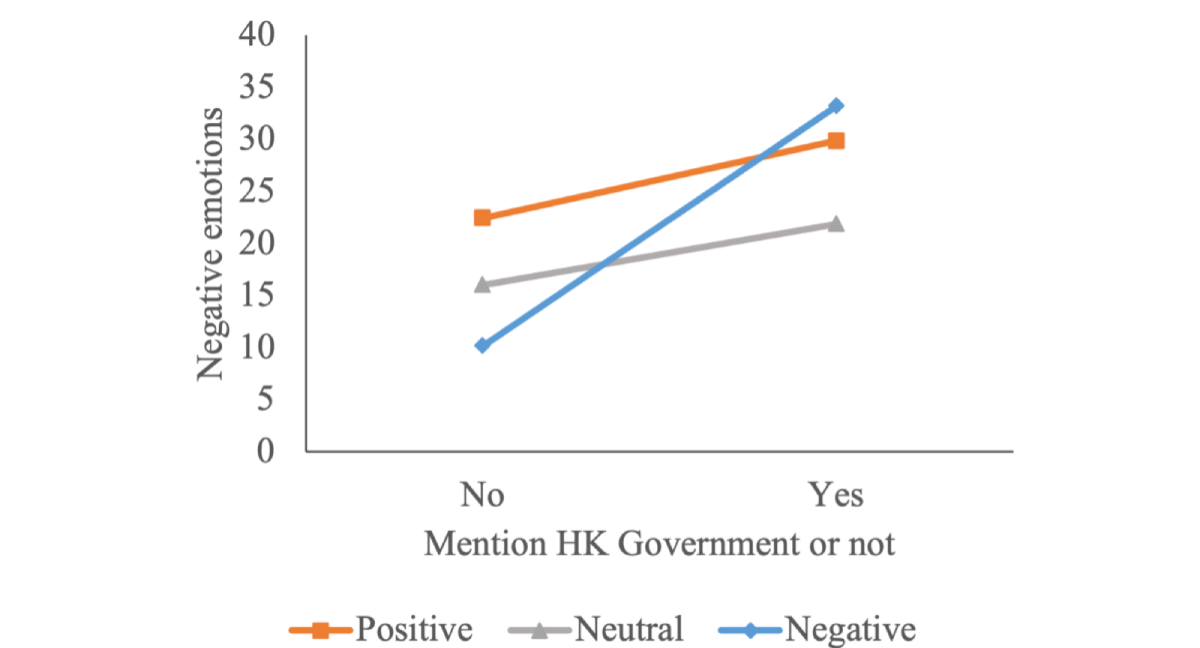
Interaction effect between media tone and occurrence of the Hong Kong (HK) Government.

**Figure 8:**
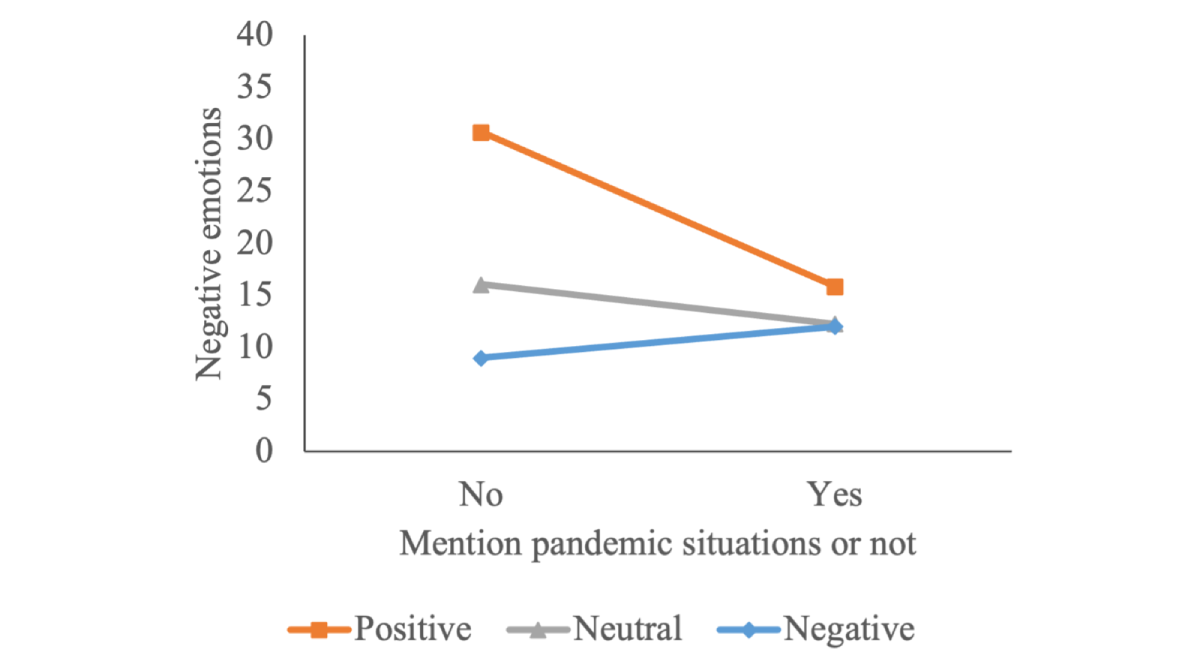
Interaction effect between media tone and occurrence of pandemic situations.

As for the interaction effects of the media tone with the subject and the issue, we paid extra attention to the representative components, the Government of Hong Kong, and the pandemic situations. In the case of a positive tone as the reference group, the post with a negative tone and mentioning the Government of Hong Kong had a statistically significant positive impact on the negative emotions (*B*=0.896; *P*<.001). The interaction effect of neutral tone and the occurrence of the Government of Hong Kong was positive as well, but it was not significant (*B*=0.027; *P*=.90). When it comes to the issue, both the negative (*B*=0.950; *P*<.001) and neutral tone (*B*=0.391; *P*=.07) of the news post had a positive interaction effect with the mention of pandemic situations, respectively, on the negative user emotions, and still, the latter was not significant enough. We plotted the interaction diagram in Figures 7 and 8 to illustrate the interaction effects straightforwardly, from which we can tell that the negative tone of the post, if meeting the subject of the Government of Hong Kong or the issue of pandemic situations, would increase the user’s negative emotions notably.

We also explored the relationship between the length of the post and its quadratic form with negative emotions separately. The length of the post had a significant negative effect on the negative emotions (*B*=–0.614; *P*=.003), while the squared term of length indicated a significant positive effect (*B*=0.651; *P*=.02). It suggested that the negative effect of post length on the count of negative emotions increases with higher values of length. Table 6 summarizes our hypothesis-testing results.

**Table 5:** Regression results^a^.

Characteristics	*Dependent variable:* negative emotions			
	Model 1, *B* (SE)	Model 2, *B* (SE)	Model 3, *B* (SE)	Model 4, *B* (SE)
Neutral tone	−0.338^b^ (0.084)	−0.339^b^ (0.094)	−0.648^c^ (0.198)	−0.662^c^ (0.205)
Negative tone	−0.496^b^ (0.103)	−0.791^b^ (0.117)	−1.228^b^ (0.227)	−1.397^b^ (0.234)
Antipandemic measures	−0.050^c^ (0.018)	−0.046^b^ (0.018)	−0.049^c^ (0.018)	−0.042^d^ (0.018)
Pandemic situations	−0.261^b^ (0.022)	−0.261^b^ (0.021)	−0.659^c^ (0.217)	−0.679^c^ (0.217)
Supportive actions	−0.083^d^ (0.035)	−0.093^c^ (0.035)	−0.087^d^ (0.035)	−0.099^c^ (0.035)
Info	0.005 (0.018)	0.008 (0.018)	0.004 (0.018)	0.010 (0.018)
Central government	0.120^b^ (0.033)	0.102^c^ (0.033)	0.119^b^ (0.033)	0.095^c^ (0.033)
HKgov^e^	0.324^b^ (0.019)	0.285 (0.209)	0.323^b^ (0.019)	0.269 (0.209)
Sector	−0.058 (0.035)	−0.055 (0.035)	−0.057 (0.035)	−0.058 (0.035)
Length	−0.166^d^ (0.081)	−0.206^d^ (0.081)	−0.175^d^ (0.081)	−0.614^c^ (0.200)
Case	0.430^b^ (0.041)	0.428^b^ (0.040)	0.430^b^ (0.041)	1.142^b^ (0.153)
Length2	N/A^f^	N/A	N/A	0.651^d^ (0.285)
Case2	N/A	N/A	N/A	−0.730^b^ (0.152)
Neutral tone:HKgov	N/A	0.027 (0.209)	N/A	0.041 (0.209)
Negative tone:HKgov	N/A	0.896^b^ (0.248)	N/A	0.815^c^ (0.249)
Neutral tone:pandemic situations	N/A	N/A	0.391 (0.218)	0.415 (0.218)
Negative tone:pandemic situations	N/A	N/A	0.950^b^ (0.255)	0.824^c^ (0.256)
Constant	2.647^b^ (0.094)	2.658^b^ (0.103)	2.966^b^ (0.201)	2.998^b^ (0.208)
Theta	0.687^b^ (0.006)	0.688^b^ (0.006)	0.688^b^ (0.006)	0.689^b^ (0.006)

^a^n=23,705 observations were used in this regression analysis. Poster=yes for all models and month=yes for all models.

^b^*P*<.001.

^c^*P*<.01.

^d^*P*<.05.

^e^HKgov: Hong Kong Government.

^f^N/A: not applicable.

**Table 6:** Summary of hypothesis-testing results.

Hypothesis	Result
1	Unsupported
2	Supported
3a	Unsupported
3b	Supported
3c	Supported
4	Supported

## Discussion

### Principal Findings

This paper systematically presents the primary concern of Hong Kong citizens amid the fifth wave of the COVID-19 pandemic. We fill the research gap by not only depicting but also excavating the factors that might explain the elicitation of negative emotions from the perspective of coverage by news organizations on social media. Besides, we use Facebook, the most widely used social platform in Hong Kong, to shed light on the negativeness of the audience and thus provide references for stakeholders to grasp public opinion, improve residents’ psychological well-being, and implement crisis communication more effectively under similar circumstances. We find that the occurrences of issues regarding pandemic situations, antipandemic measures, and supportive actions are likely to reduce the public’s negative emotions, whereas comments on the posts mentioning the central government and the Government of Hong Kong reveal more negativeness. Besides, media tone can interact with the subjects and the issues in the news to affect users’ negative emotions. Our findings about the prominent news elements are consistent with previous research studying emotional responses to news coverage by newspapers [15]. The relationship between post length and negative emotions is nonlinear. As the length of a post increases, there tends to be a decrease in the count of negative emotions experienced by individuals, but those more verbose posts will trigger more negative responses, echoing other research on article length and user engagement [55]. In the following sections, we discuss our theoretical contributions and practical implications, followed by reflections on limitations and future research.

### Theoretical Contributions

Our findings about the key elements of news coverage situate well with the appraisal theory from which we developed the main research framework. To our best knowledge, our work is the first to examine the impacts of news coverage regarding the COVID-19 pandemic on user emotions published by official news organization accounts on social media, carving out a new space for research on the emotional influences of web news coverage. Among the issues and subjects identified from scratch, we recognize the components that may either arouse or relieve the users’ negative emotions. Research regarding news coverage on the COVID-19 pandemic often places much emphasis on pandemic updates and public health efforts, which is reasonable because it is crucial to inform and educate the public under a crisis of great uncertainty [100]. Our study is consistent with previous research that mentions that issues regarding antipandemic measures could reduce negative emotional responses. Counterintuitively, the news posts updating pandemic situations are accompanied by a lower level of negative emotions. This finding may be because the public’s negative emotions are more likely to be elicited by a high level of uncertainty [48,101,102] than an informative environment. People have the innate intention to know the progress of ongoing events and need reassurance that the situation is under proper settlement [103]. Timely updates, such as the current situation, investigation of the chain of transmission, and prediction about a future trend, may reduce uncertainty, which assists people in getting over the crisis psychologically [103]. Aside from the informative coverage on combating the pandemic, we argue that supportive actions also have a place in relieving the negative emotions of the news audience, although our data reveal that the media attention assigned to this issue is not that outstanding compared with other issues. When it comes to the subjects, we find that the occurrence of political crisis managers, that is, the central government of the Chinese mainland and the Government of Hong Kong, both increase the negative emotional responses, which is in accordance with prior research on how appearances of political actors in news affect user emotionality [15,87]. The government may inevitably become the target of public censure, but there still exists ways to improve community resilience for the rehabilitation from COVID-19. The negative relationship between the occurrence of functional sectors and negative emotions, although not significant, suggests the potential capability of the efforts of the refined and specialized functional sectors to be acknowledged by the general audience. This finding about supportive actions and functional sectors might support the establishment of community resilience on social media from the angles of organizational linkages, cooperation, and collective efficacy empowerment [104,105]. Apart from the main elements of news coverage, the text length manifests a curvilinear relationship with the negative emotional responses because of the possible cognitive overload. The message may be informative with the increased length at the beginning but add unnecessary load later, suggesting that the news length is not supposed to be merely a control variable as in previous research but an element to be considered in analyzing news coverage.

In addition, we contribute to expanding research on the priming effects of media tone. Our results concerning interaction effects demonstrate that the negative media tone, when co-occurring with the representative issue and subject, that is, the Government of Hong Kong and the pandemic situation, would be more likely to ignite negative emotions in user commenting. However, the main effect of the negative and neutral media tone would decrease the negative emotions compared with the positive media tone. The backfire effects might explain this finding because the backfiring may occur in the audience’s understanding process involved in forced consensus [97,106], and the positive tone in a nonoptimistic crisis environment might induce unanticipated and undesirable consequences.

### Practical Implications

Our work also provides practical implications from 3 perspectives. First, we suggest crisis communication strategies for the government to play a better role as a crisis manager to fulfill the public’s expectations and thus reduce negative emotions and boost community resilience. A crisis might be a turning point for better or worse, in which shrewd crisis management plays a key role [107]. The public’s disappointment at the government is likely to rise when the latter part’s promises fail to be fulfilled, yet the flexibility and transparency in crisis communication are effective in dealing with uncertainty and a lack of trust, especially during the unexpected burst of pandemics [108]. Transparency, although likely to initially cause uneasiness, fear, and distrust, eventually would contribute to regaining public trust and reduce redundant panic in the long term [109,110]. Meanwhile, it is noteworthy that functional sectors deserve much more attention. As community resilience increasingly enters the view of the public and administrative sectors, it is vital to engage local people in the entire process of mitigation, build organizational connections, and preserve social supports [104]. Emphasis on the existence of these specialized functional sectors might be a shot in the arm for the restless people.

Moreover, our study offers suggestions for news organizations on coverage in social media to improve the audience’s psychological well-being and maintain a harmonious web-based public opinion environment. Our work suggests 2 criteria in view of the news coverage components that reduce negative emotions on social media. The first is extra immediacy, which is essential for mass communication amid the pandemic [111]. The issues of antipandemic measures and pandemic situations both require up-to-date messages and quick responses in terms of those constantly updating information [112]. The timely essence of the measures coping with the COVID-19 pandemic puts forth the same pace as the news coverage [113]. Another criterion is effectiveness, which ensures proper information management across the stages of a pandemic. Public health efforts to keep an outbreak under control and update on confirmed cases usually occupy people’s attention during the initial stages of a pandemic. However, when the public has better knowledge as the pandemic unfolds, additional information is anticipated [100], in which case the previous pattern of coverage is no longer satisfactory. Issues such as supportive actions are able to convey collective efficacy, which signifies the postcrisis period and is beneficial to rebuilding community resilience [105]. News organizations should also be careful that an excessively detailed news post on social media is not necessarily the most effective way to be informative. Endeavors aligning with these findings may reduce the elicited user emotions when they rely on social media for news consumption and consequently facilitate a harmonious web environment [114].

Last but not least, through an examination of the emotional responses elicited by news coverage by news organizations on social media, the findings of this study provide insights into the possible change in attitudes toward health behavior, considering the role of emotions in attitudinal change [115]. Individuals may be accommodated to be more receptive to health-related information conveyed through news reports with strategies that avoid negative emotional stimulators.

### Conclusions

Understanding the relationship between the negative emotions of commenters and news coverage on social media is of great significance not only for prompting rapid and efficient public health responses but also for shaping and maintaining individuals’ values. By using machine learning techniques, this study sheds light on the impacts of various components of news coverage (issues, subjects, media tone, and length) on social media on the public’s negative emotions (anger, fear, and sadness). Our comprehensive analysis culminates in results that will provide a reference framework for efficient crisis communication in the case of similar pandemics at present or in the future. This research, although discovering new findings by first extending the analysis between the components of news coverage and negative user emotions to the scenario of social media, echoes previous studies drawn from traditional media and its derivatives, such as web newspapers. Although the era of the COVID-19 pandemic gradually brings down the curtain, the commonality of this research and previous studies also contributes to establishing clearer territory in the field of health crises.

Besides the aforementioned theoretical and practical implications, this study still has several limitations. First, even though we applied state-of-the-art machine learning techniques to reveal emotion dynamics and measure the media tone, it is unavoidable that misclassifications still occur in text processing procedures. BERT-based models are among the top natural language processing models for emotion and sentiment analysis, but as with other machine learning models, they cannot achieve 100% accuracy, such as the difficulty in understanding informal language, which can be quite common in web-based communication and social media to express their emotions [116]. The misclassifications may be unavoidable in research involving automatic detection with large-scale data, and continuous improvement and customization are needed in future research [78]. Future research may apply methods to address this prevalent problem caused by using machine learning classification outcome as a variable in regression [117]. Besides, although we control the individual fixed effects of the poster as well as the time fixed effects, and most of the effects in our model are significant, our findings unravel the correlations of variables instead of causal relationships. We plan to explore more causality between news coverage and user emotions in future research. In addition, the research context of our study is limited to Hong Kong in the context of the fifth wave of the COVID-19 pandemic and Facebook due to the representativeness of the platform and the pandemic situation. Hence, we acknowledge that the findings are not necessarily generalizable to other countries, other platforms, or other waves of the COVID-19 pandemic in Hong Kong. Although we fill the research gap in revealing and explaining the emotion dynamics of the fifth wave of the COVID-19 pandemic through the lens of news coverage on social media, we welcome further evidence to prove its adaptability from diverse perspectives.

## Appendix

Multimedia Appendix 1 Descriptive details of materials and analysis.

## Abbreviations

BERT: Bidirectional Encoder Representations from Transformers
RQ: research question

## Data Availability

The data sets generated during and analyzed during this study are available from the corresponding author on reasonable request.
