# Supplementary material for: News Coverage of the COVID-19 Pandemic on Social Media and the Public’s Negative Emotions: Computational Study
Source: J Med Internet Res. 2024 Jun 6;26:e48491. doi: 10.2196/48491 (PMC11190626; doi:10.2196/48491)
Supplement: Multimedia Appendix 1 [file jmir_v26i1e48491_app1.doc]

**Multimedia Appendix 1**

**Table S1. Public pages of the major news organizations**

|  | Public pages |
| --- | --- |
| 1 | 香港電台視像新聞 RTHK VNEWS <https://www.facebook.com/RTHKVNEWS/> |
| 2 | 香港警察 Hong Kong Police <https://www.facebook.com/HongKongPoliceForce/> |
| 3 | 香港經濟日報 hket.com <https://www.facebook.com/hketpage/> |
| 4 | 香港獨立媒體網 <https://www.facebook.com/inmediahknet/> |
| 5 | 香港商報 <https://www.facebook.com/news.hkcd/> |
| 6 | 新華香港 <https://www.facebook.com/XinhuaHK/> |
| 7 | RTHK 香港電台 <https://www.facebook.com/RTHK.HK/> |
| 8 | 明報即時新聞 <https://www.facebook.com/mingpaoinews/> |
| 9 | 眾新聞 <https://www.facebook.com/hkcnews/> |
| 10 | Now News – 新聞 <https://www.facebook.com/now.comNews/> |
| 11 | 01新聞 <https://www.facebook.com/hk01.news/> |
| 12 | 信報財經新聞 hkej.com <https://www.facebook.com/hongkongeconomicjournal/> |
| 13 | 突發最前線 [https://www.facebook.com/%E7%AA%81%E7%99%BC%E6%9C%80%E5%89%8D%E7%B7%9A-1672730629675861/](https://www.facebook.com/突發最前線-1672730629675861/) |
| 14 | 政府新聞網 <https://www.facebook.com/govnews.hk/> |
| 15 | 香港文匯報 <https://www.facebook.com/wenweipo/> |
| 16 | 大公報 – 大公網 [https://www.facebook.com/%E5%A4%A7%E5%85%AC%E5%A0%B1-%E5%A4%A7%E5%85%AC%E7%B6%B2-1693275180942630/](https://www.facebook.com/大公報-大公網-1693275180942630/) |
| 17 | 星島日報 [https://www.facebook.com/%E6%98%9F%E5%B3%B6%E6%97%A5%E5%A0%B1-178766115480756/](https://www.facebook.com/星島日報-178766115480756/) |
| 18 | 有線新聞 i-Cable News  <https://www.facebook.com/icablenews/> |
| 19 | 東方日報 [https://www.facebook.com/%E6%9D%B1%E6%96%B9%E6%97%A5%E5%A0%B1-638924839545704/](https://www.facebook.com/東方日報-638924839545704/) |
| 20 | on.cc 東網／東方日報   <https://www.facebook.com/onccnews/> |
| 21 | AM730 <https://www.facebook.com/am730hk/> |
| 22 | 端傳媒 <https://www.facebook.com/theinitium/> |
| 23 | HKG報 <https://www.facebook.com/hkgpaocomhk/> |
| 24 | Bastillepost 巴士的報 <https://www.facebook.com/Bastillepost/> |
| 25 | TOPick 新聞 <https://www.facebook.com/topick.hket/> |
| 26 | 晴報 Sky Post <https://www.facebook.com/Skyposthk/> |
| 27 | PassionTimes 熱血時報 <https://www.facebook.com/passiontimes/> |

Table S2. Discovered topics with top words in each topic.

| Topic ID | Topic name | Top words in topic |
| --- | --- | --- |
| 1 | Regional mandatory testing | test (檢測), mandatory (強制), government (政府), personage (人士), citizens (居民), district (區域), Kuiyong (葵涌邨), accept (接受) |
| 2 | Anti-epidemic work Instructions from the central government | epidemic situation (疫情), anti-pandemic (抗疫), health (健康), work (工作), mainland (內地), central (中央), government (政府), economics (經濟), Shanghai (上海), special zone (特區) |
| 3 | Confirmed cases | cases (個案), epidemic situation (疫情), confirmed (確診), newly added (新增), health (衛生), live broadcast (直播), Hong Kong (本港), infection (感染) |
| 4 | Vaccination | vaccine (疫苗), inoculation (接種), hospital (醫院), Centre for Health Protection (衛生中心), patients (病人), pass (通行) |
| 5 | Epidemic situation | epidemic situation (疫情), latest (最快), call (致電), receive (接收), alarm (警報), immediately (立刻), society (社會), confirmed (確診), information (資訊) |
| 6 | Integrated news | track (追蹤), epidemic situation (疫情), police (警方), brand (品牌), America (美國), promotion (推介), Shanghai (上海), Russia (俄羅斯) |
| 7 | Anti-epidemic measures by Government of Hong Kong | epidemic situation (疫情), government (政府), Carrie Lam (林鄭月娥), measure (措施), test (檢測), universal (全民), citizens (市民), anti-pandemic (防疫) |
| 8 | Supportive actions | support (支持), isolation (隔離), facility (設施), sponsor (贊助), Lee Ka-chiu (李家超), Chief executive (特首), work (工作), plan (計劃), host (主持), independent (獨立) |

Table S3. Variance inflation factors.

| Variable | GVIF | Df | GVIF^(1/(2*Df)) |
| --- | --- | --- | --- |
| Sentiment | 1.078494 | 2 | 1.019509 |
| Anti | 1.211011 | 1 | 1.100460 |
| Situation | 1.141843 | 1 | 1.068570 |
| Supp | 1.051323 | 1 | 1.025340 |
| Info | 1.162401 | 1 | 1.078147 |
| Central | 1.105374 | 1 | 1.051368 |
| HKgov | 1.256599 | 1 | 1.120981 |
| Sector | 1.146691 | 1 | 1.070836 |
| Length | 1.778414 | 1 | 1.333572 |
| Case | 1.017428 | 1 | 1.008677 |
| Account | 2.371008 | 25 | 1.017416 |
